# Supplementary material for: Phenolic compounds induce ferroptosis-like death by promoting hydroxyl radical generation in the Fenton reaction
Source: Commun Biol. 2024 Feb 17;7:199. doi: 10.1038/s42003-024-05903-5 (PMC10874397; doi:10.1038/s42003-024-05903-5)
Supplement: Supplementary file 2 — Supplementary Information [file 42003_2024_5903_MOESM2_ESM.pdf]

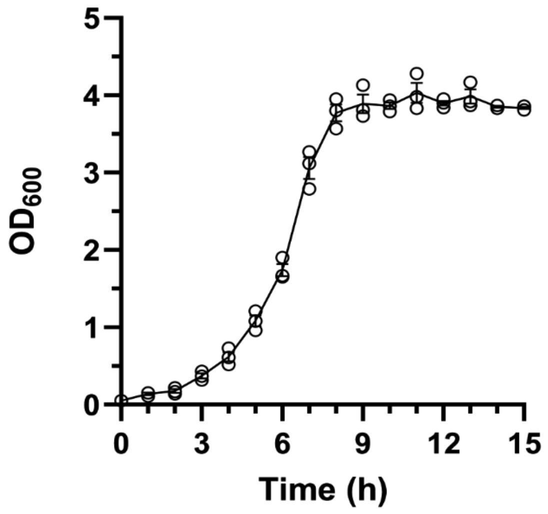

Supplementary Figure 1 Growth curve of *E. coli* BL21(DE3) in minimal salt medium. $n = 3$ biological independent samples. Error bar, mean ± SEM.

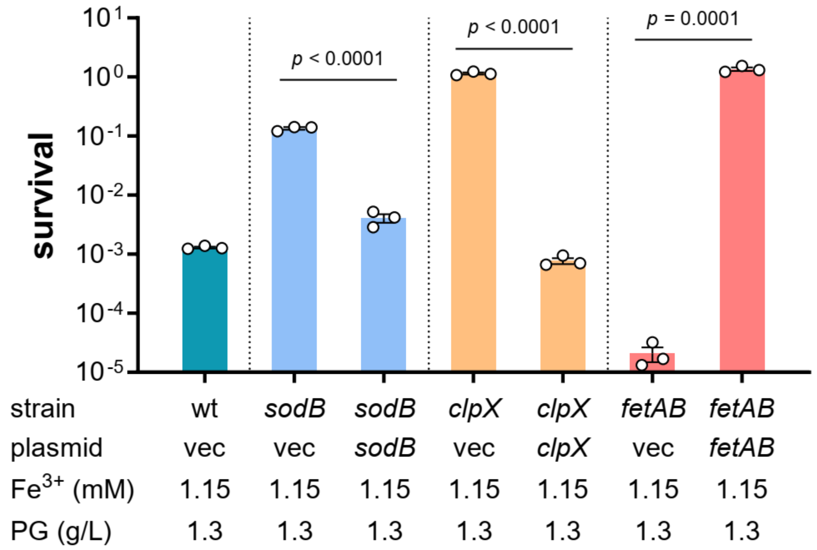

Supplementary Figure 2 Confirmation of the role of *sodB*, *clpX*, and *fetAB* in PG tolerance by knockout and complementation. $n = 3$ biological independent samples. Error bar, mean ± SEM.

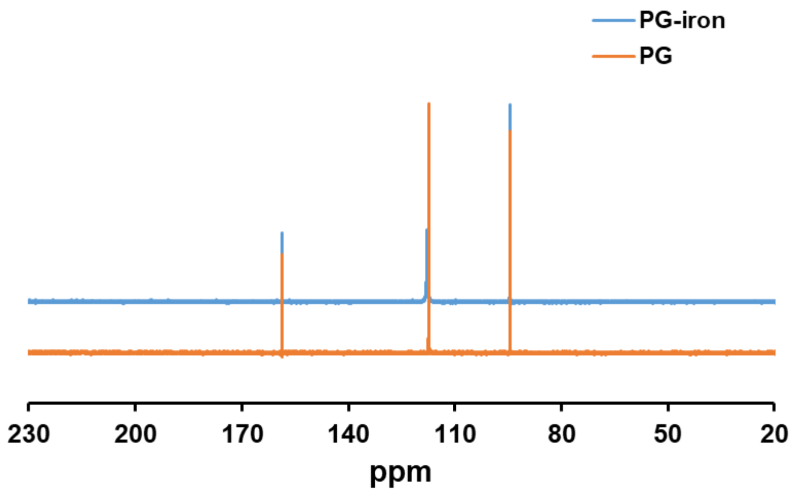

Supplementary Figure 3 ^{13}C NMR of PG and PG-iron complex.

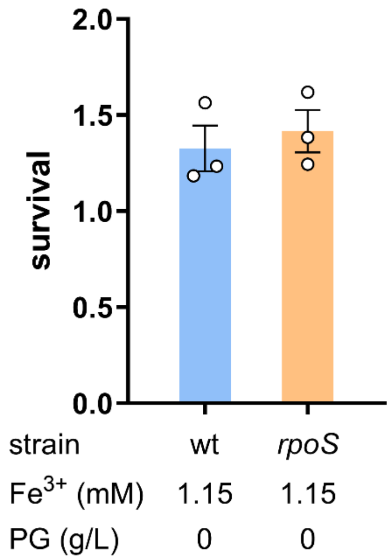

Supplementary Figure 4 Survival of *E. coli* wild-type and *rpoS* mutant without the presence of PG. *n* = 3 biological independent samples. Error bar, mean ± SEM.

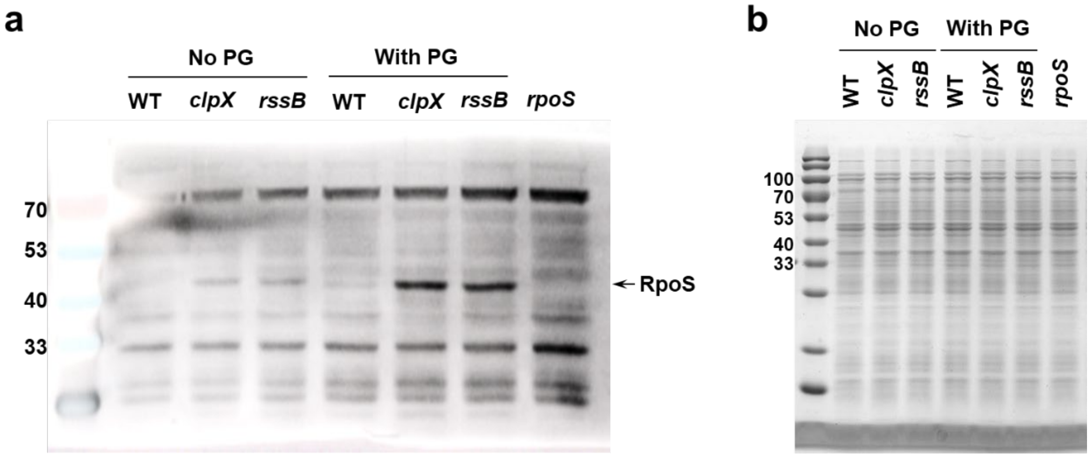

Supplementary Figure 5 Original image of RpoS Western blot shown in Fig. 4c (a) and SDS-PAGE (b).

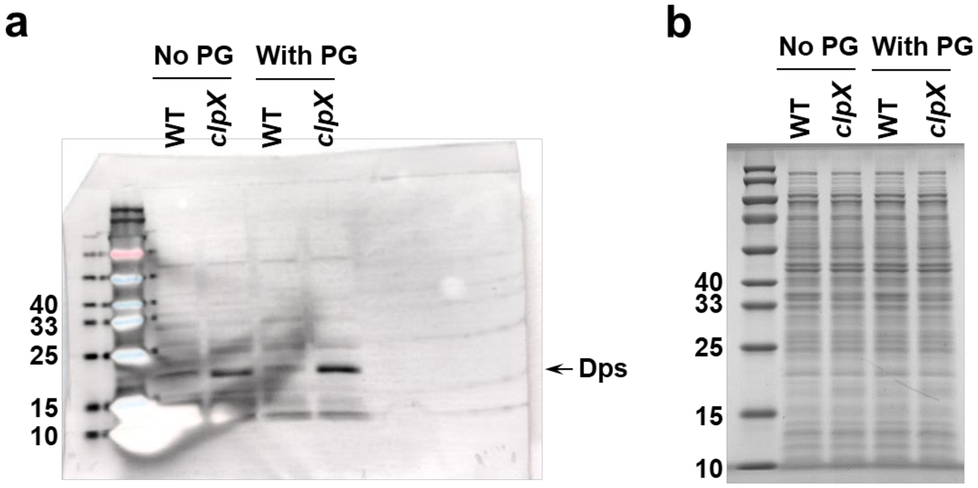

Supplementary Figure 6 Original image of Dps-his$_6$ Western blot shown in Fig. 4e (a) and SDS-PAGE (b).

```
Human_GPX4      MSLGRLCRLLKPALLCGALAAPGLAGTMCASRDDWRCARSMHEFSAKDIDGHMVNLDKYR      60
Ecoli_BtuE      ------------------------------------MQDSILTTVVKDIDGEVTTLEKFA      24
                                                       *:    .*****.:..*:*:

Human_GPX4      GFVCIVTNVASQUGKTEVNYTQLVDLHARYAECGLRILAFPCNQFGKQEPGSNEEIKEFA      120
Ecoli_BtuE      GNVLLIVNVASKCGLT-PQYEQLENIQKAWVDRGFMVLGFPCNQFLEQEPGSDEEIKTYC      83
                * * ::.****: * *  :* ** :::  :.: *: :*.****** :*****:**** :.

Human_GPX4      -AGYNVKFDMFSKICVNGDDAHPLWKWMKIQ------PK-----------G-KGILGNAI      161
Ecoli_BtuE      TTTWGVTFPMFSKIEVNGEGRHPLYQKLIAAAPTAVAPEESGFYARMVSKGRAPLYPDDI      143
                 : :.*.* ***** ***:.  ***:: :         *:            *   :  : *

Human_GPX4      KWNFTKFLIDKNGCVVKRYGPMEEP---LVIEKDLPHYF-      197
Ecoli_BtuE      LWNFEKFLVGRDGKVIQRFSPDMTPEDPIVMESIKLALAK      183
                 *** ***:.::* *::*:.*   *   :*:*.
```

Supplementary Figure 7 Sequence alignment of human GPX4 and *E. coli* BtuE proteins. The protein sequences used here are under the Accession Numbers WP_001154168.1 and NP_416225.1.

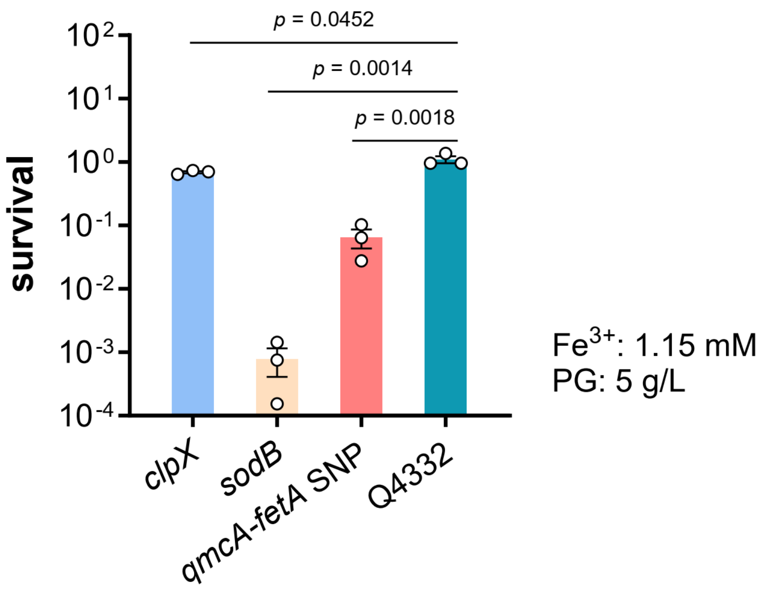

Supplementary Figure 8 Survival rates of *E. coli* BL21(DE3) mutants upon exposure to 5 g/L PG (n = 3 biological independent samples). Error bar, mean ± SEM.

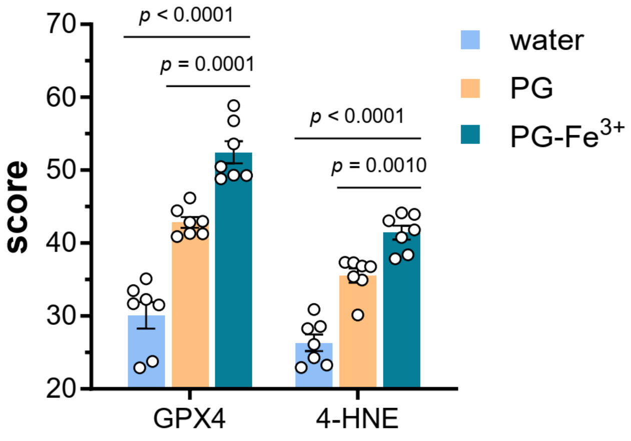

Supplementary Figure 9 IHC results from H1299 tumors in mice treated with water, PG and PG-iron complex. Glutathione peroxidase 4 (GPX4) and 4-hydroxynonenal (4-HNE) were detected using corresponding antibodies. The ImageJ software was used to quantify the images. $n = 7$ biological independent samples. Error bar, mean ± SEM.

Supplementary Table 1 Identified mutations arising during ALE in the evolved strains

location[a]	gene	mutation	M01	M02	M03	M04	M05	M06
54709	*apaH*	P237Q	√	√	√	√	√	√
108750	*ftsZ*	R214H	√	√	√			
171290	*fhuA*	E322*, truncated				√	√	√
172410	*fhuA*	Δ 1 bp, frameshift	√	√	√			
425702	*clpX*	8 bp insertion, frameshift	√	√	√			
425705	*clpX*	Δ 13 bp, frameshift	√	√	√			
425720	*clpX*	6 bp in-frame insertion	√	√	√			
425725	*clpX*	G206R		√	√			
425728	*clpX*	3 bp in-frame insertion	√					
425730	*clpX*	A→T, synonymous variant	√					
425732	*clpX*	Q208P			√			
425736	*clpX*	2 bp insertion, frameshift	√					
425737	*clpX*	3 bp in-frame insertion	√					
485006	*qmcA-fetA*	C→G, intergenic region				√	√	√
749930	*lacI-lacZ*	113 bp insertion, *lacZ* is a pseudogene disrupted by prophage DE3						√
771458	*B*	72 bp insertion, truncated, λ phage protein	√	√	√	√		√
1141896	*flgK*	71 bp insertion, frameshift		√			√	
1141897	*flgK*	66 bp insertion					√	
1681002	*sodB*	Δ 21 bp, T147-L153 deleted	√	√	√	√	√	√
2402949	*zipA*	C→A, synonymous variant	√					
3219144	*arcZ*	1 bp insertion, frameshift	√	√	√			
3406753	*nfuA-gntT*	C→T, intergenic region	√	√	√			
3822342	*rbsD*	61 bp insertion, pseudogene	√		√			
3822349	*rbsD*	G→C, pseudogene	√		√			

3822352	*rbsD*	C→T, pseudogene			√			
3887966	*yigB*	Δ 1 bp, frameshift				√	√	√
3960500	*yshB-glnG*	G→T, intergenic region	√	√	√			
4450213	*insB27*	G→T, pseudogene				√		

[a] according to the reference sequence of *E. coli* BL21(DE3) strain (NC_012978.2).

Supplementary Table 2 Bacterial strains and plasmids used in this study

Strain or plasmid	Description	Source
***E. coli* strains**		
DH5α	F^- *supE*44 Δ*lacU*169 (ϕ80 *lacZ* Δ*M15*) *hsdR*17 *recA*1 *endA1* *gyrA*96 *thi*-1 *relA*1	Lab collection
BL21(DE3)	F^- *ompT gal dcm lon hsdSB* (rB^- mB^-) λ(DE3)	Lab collection
M01	BL21(DE3) evolved strain No. 1	This study
M02	BL21(DE3) evolved strain No. 2	This study
M03	BL21(DE3) evolved strain No. 3	This study
M04	BL21(DE3) evolved strain No. 4	This study
M05	BL21(DE3) evolved strain No. 5	This study
M06	BL21(DE3) evolved strain No. 6	This study
Q3497	BL21(DE3) Δ*fhuA*	This study
Q3498	BL21(DE3) Δ*yigB*	This study
Q3499	BL21(DE3) Δ*sodB*	This study
Q3500	BL21(DE3) Δ*flgK*	This study
Q3501	BL21(DE3) Δ*clpX*	This study
Q3665	BL21(DE3) Δ*rpoS*	This study
Q3671	BL21(DE3) Δ*arcZ*	This study
Q3672	BL21(DE3) Δ*rssB*	This study
Q3850	BL21(DE3) Δ*sodA*	This study
Q3851	BL21(DE3) Δ*sodC*	This study
Q3852	BL21(DE3) Δ*katG*	This study
Q3853	BL21(DE3) Δ*katE*	This study
Q5634	BL21(DE3) Δ*fetAB*	This study
Q5638	BL21(DE3) ΔkatG ΔkatE	This study
Q3854	BL21(DE3) $apaH^{P237Q}$ (54709 G to T)	This study
Q3855	BL21(DE3) *qmcA-fetA* SNP (485006 C to G)	This study
Q3856	BL21(DE3) $ftsZ^{R214H}$ (108750 G to A)	This study

Q3857	BL21(DE3) *yshB-glnG* SNP (3960500 G to T)	This study
Q4343	BL21(DE3) *nfuA-gntT* SNP (3406753 C to T)	This study
Q4332	BL21(DE3) Δ*clpX* Δ*sodB qmcA-fetA* SNP	This study
Q4340	BL21(DE3) Δ*clpX*Δ*rpoS*	This study
Q5639	BL21(DE3) *dps-his*$_6$	This study
Q5640	BL21(DE3) Δ*clpX dps-his*$_6$	This study
Q5641	BL21(DE3) Δ*clpX* / pTrcHis2B	This study
Q5642	BL21(DE3) Δ*sodB* / pTrcHis2B	This study
Q5643	BL21(DE3) Δ*fetAB* / pTrcHis2B	This study
Q5635	BL21(DE3) Δ*clpX* / pTRC-*clpX*	This study
Q5636	BL21(DE3) Δ*sodB* / pTRC-*sodB*	This study
Q5637	BL21(DE3) Δ*fetAB* / pTRC-*fetAB*	This study
Q3595	BL21(DE3)/pA-*phlD*/*marA*/*acc*	[1]
Q4333	Q4332/pA-*phlD*/*marA*/*acc*	This study
Q1013	*Salmonella typhimurium* LT2	CGMCC[a]
Q1188	*Klebsiella pneumoniae* ATCC25955	CICC[b]
Q4347	*Pseudomonas* sp. DHS3Y	CICC[b]
Q1915	*Saccharomyces cerevisiae* s288c	Lab stock
**Plasmids**		
pTrcHis2B	rep_{pBR322} Amp^R *lacI*^{q} P_{trc}	Invitrogen
pET28a	rep_{pBR322} Kan^R *lacI*^{q} P_{T7}	Invitrogen
pTargetF	rep_{pMB1} $Spec^R$ J23119-sgRNA	MolecularCloud: MC0000012[2]
pCas	$rep_{pSC101^{Ts}}$ Kan^R P_{cas}-*cas9* P_{araB}-Red *lacI*^{Q} P_{trc}-$sgRNA_{pMB1}$	MolecularCloud: MC0000011[2]
pKSI-1	Amp^R pBluescript II KS (-) backbone with I-*Sce*I site–multiple-cloning sites–I-*Sce*I site cassette	Addgene: #51725[3]
pREDTKI	$rep_{pSC101^{Ts}}$ Kan^R P_{cas}-*cas9* P_{araB}-Red *lacI*^{Q} P_{trc} I-*Sce*I site	Addgene: #51628[3]

pMDIAI	AprR AmpR pMD18-T backbone; apramycin resistance gene flanked by FRT and I-*Sce*I sites	Addgene: #51655[3]
pA-p*hlD*/*marA*/*acc*	rep_{p15A} Cm^R *lacI* P_{T7}-*phlD-marA-accADBC*	[1]
pQ-lib	a random mutant library of *dnaQ* gene of *E. coli* carried by the pUC18 vector	[4]
pTB*clpX*	rep_{pMB1} $Spec^R$ J23119-sgRNA$_{clpX}$ Δ*clpX*::Not I	This study
pTB*rssB*	rep_{pMB1} $Spec^R$ J23119-sgRNA$_{rssB}$ Δ*rssB*::Not I	This study
pTB*sodB*	rep_{pMB1} $Spec^R$ J23119-sgRNA$_{sodB}$ Δ*sodB*::Not I	This study
pTB*arcZ*	rep_{pMB1} $Spec^R$ J23119-sgRNA$_{arcZ}$ Δ*arcZ*::Not I	This study
pTB*rpoS*	rep_{pMB1} $Spec^R$ J23119-sgRNA$_{rpoS}$ Δ*rpoS*::Not I	This study
pTB*fhuA*	rep_{pMB1} $Spec^R$ J23119-sgRNA$_{fhuA}$ Δ*fhuA*::Not I	This study
pTB*flgK*	rep_{pMB1} $Spec^R$ J23119-sgRNA$_{flgK}$ Δ*flgK*::Not I	This study
pTB*yigB*	rep_{pMB1} $Spec^R$ J23119-sgRNA$_{yigB}$ Δ*fhuA*::Not I	This study
pTB*sodA*	rep_{pMB1} $Spec^R$ J23119-sgRNA$_{sodA}$ Δ*sodA*::Not I	This study
pTB*sodC*	rep_{pMB1} $Spec^R$ J23119-sgRNA$_{sodC}$ Δ*sodC*::Not I	This study
pTBftsZ	rep_{pMB1} $Spec^R$ J23119-sgRNA$_{ftsZ}$ Δ*ftsZ*::IS-Apm^R-IS	This study
pKSIMB*ftsZ*	Amp^R Donor plasmid for *ftsZ*^{R214H}	This study
pTB*fetA*	rep_{pMB1} $Spec^R$ J23119-sgRNA$_{fetA}$ Δ*fetA*::IS-Apm^R-IS	This study
pKSIMB*fetA*	Amp^R Donor plasmid for *qmcA-fetA* SNP	This study
pTB*apaH*	rep_{pMB1} $Spec^R$ J23119-sgRNA$_{apaH}$ Δ*apaH*::IS-Apm^R-IS	This study
pKSIMB*apaH*	Amp^R Donor plasmid for *apaH*^{P237Q}	This study
pTB*yshB*	rep_{pMB1} $Spec^R$ J23119-sgRNA$_{yshB}$ Δ*yshB*::IS-Apm^R-IS	This study
pKSIMB*yshB*	Amp^R Donor plasmid for *yshB-glnG* SNP	This study
pTB*gntT*	rep_{pMB1} $Spec^R$ J23119-sgRNA$_{gntT}$ Δ*gntT*::IS-Apm^R-IS	This study
pKSIMB*gntT*	Amp^R Donor plasmid for *nfuA-gntT* SNP	This study
pTRC-*qmcA*	rep_{pBR322} Ap^R *lacI*^{q} P_{trc}-*qmcA*	This study
pTRC-*fetAB*	rep_{pBR322} Ap^R *lacI*^{q} P_{trc}-*fetAB*	This study
pTRC-*katG*	rep_{pBR322} Ap^R *lacI*^{q} P_{trc}-*katG*	This study
pTRC-*katE*	rep_{pBR322} Ap^R *lacI*^{q} P_{trc}-*katE*	This study

pTRC-*btuE*	rep$_{pBR322}$ Ap^R *lacI*^{q} P$_{trc}$-*btuE*	This study
pET28a-*rpoS*	rep$_{pBR322}$ Kan^R *lacI*^{q} P$_{T7}$-*rpoS*	This study
pTRC-*sodB*	rep$_{pBR322}$ Ap^R *lacI*^{q} P$_{trc}$-*sodB*	This study
pTRC-*clpX*	rep$_{pBR322}$ Ap^R *lacI*^{q} P$_{trc}$-*clpX*	This study
pPaper-Δ*fetAB*	p15A Cm^R P$_{lacIQ}$-sgRNA-Tet^R J23119-sgRNA Δ*fetAB*	This study
pPaper-*dps$_{his}$*	p15A Cm^R P$_{lacIQ}$-sgRNA-Tet^R J23119-sgRNA *dps$_{his}$*	This study
pTB*katG*	rep$_{pMB1}$ Spec^R J23119-sgRNA$_{clpX}$ Δ*katG*::NotI	This study
pTB*katE*	rep$_{pMB1}$ Spec^R J23119-sgRNA$_{clpX}$ Δ*katE*::NotI	This study

[a] China General Microbiological Culture Collection Center

[b] China Center of Industrial Culture Collection

Supplementary References

1. Cao YJ, Jiang XL, Zhang RB, Xian M. Improved phloroglucinol production by metabolically engineered *Escherichia coli*. *Appl Microbiol Biotechnol* **91**, 1545-1552 (2011).

2. Jiang Y, Chen B, Duan C, Sun B, Yang J, Yang S. Multigene editing in the *Escherichia coli* genome via the CRISPR-Cas9 system. *Appl Environ Microbiol* **81**, 2506-2514 (2015).

3. Yang J*, et al.* High-efficiency scarless genetic modification in *Escherichia coli* by using lambda red recombination and I-SceI cleavage. *Appl Environ Microbiol* **80**, 3826-3834 (2014).

4. Luan G, Cai Z, Li Y, Ma Y. Genome replication engineering assisted continuous evolution (GREACE) to improve microbial tolerance for biofuels production. *Biotechnol Biofuels* **6**, 137 (2013).
