# Supplementary material for: Phenolic compounds induce ferroptosis-like death by promoting hydroxyl radical generation in the Fenton reaction
Source: Commun Biol. 2024 Feb 17;7:199. doi: 10.1038/s42003-024-05903-5 (PMC10874397; doi:10.1038/s42003-024-05903-5)
Supplement: Supplementary file 6 — Reporting Summary [file 42003_2024_5903_MOESM6_ESM.pdf]

nature portfolio

Corresponding author(s): Guang Zhao

Last updated by author(s): Jan 30, 2024

Reporting Summary

Nature Portfolio wishes to improve the reproducibility of the work that we publish. This form provides structure for consistency and transparency in reporting. For further information on Nature Portfolio policies, see our Editorial Policies and the Editorial Policy Checklist.

Statistics

For all statistical analyses, confirm that the following items are present in the figure legend, table legend, main text, or Methods section.

n/a	Confirmed	
☐	☒	The exact sample size (*n*) for each experimental group/condition, given as a discrete number and unit of measurement
☐	☒	A statement on whether measurements were taken from distinct samples or whether the same sample was measured repeatedly
☐	☒	The statistical test(s) used AND whether they are one- or two-sided<br>*Only common tests should be described solely by name; describe more complex techniques in the Methods section.*
☒	☐	A description of all covariates tested
☒	☐	A description of any assumptions or corrections, such as tests of normality and adjustment for multiple comparisons
☐	☒	A full description of the statistical parameters including central tendency (e.g. means) or other basic estimates (e.g. regression coefficient) AND variation (e.g. standard deviation) or associated estimates of uncertainty (e.g. confidence intervals)
☐	☒	For null hypothesis testing, the test statistic (e.g. *F*, *t*, *r*) with confidence intervals, effect sizes, degrees of freedom and *P* value noted<br>*Give P values as exact values whenever suitable.*
☒	☐	For Bayesian analysis, information on the choice of priors and Markov chain Monte Carlo settings
☒	☐	For hierarchical and complex designs, identification of the appropriate level for tests and full reporting of outcomes
☒	☐	Estimates of effect sizes (e.g. Cohen's *d*, Pearson's *r*), indicating how they were calculated

Our web collection on statistics for biologists contains articles on many of the points above.

Software and code

Policy information about availability of computer code

Data collection	Applied Biosystems QuantStudio 1 (Applied Biosystems) for qRT-PCR; U-2900 Spectrophotometer (Hitachi, Japan) for UV-visible absorption spectra; Avance Neo 600 NMR spectrometer (Bruker, Switzerland) for 1H and 13C NMR spectra; Eclipse E100 microscope (Nikon, Japan) for immunohistochemistry analysis; Fusion FX6 Imaging System (Vilber, France) for immunoblot imaging.
Data analysis	Data were analyzed using Microsoft Excel 2019 and plotted using Graphpad Prism 10 (Graphpad). The relative amount of each band in Western blot is determined using ImageJ (NIH).

For manuscripts utilizing custom algorithms or software that are central to the research but not yet described in published literature, software must be made available to editors and reviewers. We strongly encourage code deposition in a community repository (e.g. GitHub). See the Nature Portfolio guidelines for submitting code & software for further information.

Data

Policy information about availability of data

All manuscripts must include a data availability statement. This statement should provide the following information, where applicable:

- Accession codes, unique identifiers, or web links for publicly available datasets
- A description of any restrictions on data availability
- For clinical datasets or third party data, please ensure that the statement adheres to our policy

Source data are provided with this paper. The mutations identified, the used strains, plasmids and primers were provided in the supplementary information.

Research involving human participants, their data, or biological material

Policy information about studies with human participants or human data. See also policy information about sex, gender (identity/presentation), and sexual orientation and race, ethnicity and racism.

Reporting on sex and gender	N/A
Reporting on race, ethnicity, or other socially relevant groupings	N/A
Population characteristics	N/A
Recruitment	N/A
Ethics oversight	N/A

Note that full information on the approval of the study protocol must also be provided in the manuscript.

Field-specific reporting

Please select the one below that is the best fit for your research. If you are not sure, read the appropriate sections before making your selection.

☒ Life sciences ☐ Behavioural & social sciences ☐ Ecological, evolutionary & environmental sciences

For a reference copy of the document with all sections, see nature.com/documents/nr-reporting-summary-flat.pdf

Life sciences study design

All studies must disclose on these points even when the disclosure is negative.

Sample size	The strain growth assay, phenols challenge experiments, enzyme activity assay, phloroglucinol biosynthesis, phenol degradation, decolorization of malachite green were performed at least three replicates. Three biological independent samples with two technical repeats for each sample were used for qRT-PCR. Seven biological independent samples were used for in vivo tumor growth and treatment. Sample size was determined based on the previous experience and the majority of other metabolic engineering publications. It is sufficient to confirm that results did not vary and were consistent.
Data exclusions	No data were excluded for the analyses.
Replication	All the biochemical and biological experiments were performed at least twice, with ability to obtain similar results.
Randomization	The samples of bacterial cultures that were split into different conditions were random samplings, and there is no control over which cells will be selected. And pipet tips were used to scoop few cells for culturing, the scooping locations are all random.
Blinding	Blinding is not relevant to our study because none of our data is based on qualitative scoring metrics nor does it involve animals or human research participants. As described in the above section for randomization, blinding during group allocation is irrelevant because the samples of bacterial cultures that were split into different conditions were random samplings and there is no control over which cells will be selected and thus, no bias during group allocation.

Reporting for specific materials, systems and methods

We require information from authors about some types of materials, experimental systems and methods used in many studies. Here, indicate whether each material, system or method listed is relevant to your study. If you are not sure if a list item applies to your research, read the appropriate section before selecting a response.

Materials & experimental systems

n/a	Involved in the study	
☐	☒	Antibodies
☐	☒	Eukaryotic cell lines
☒	☐	Palaeontology and archaeology
☐	☒	Animals and other organisms
☒	☐	Clinical data
☒	☐	Dual use research of concern
☒	☐	Plants

Methods

n/a	Involved in the study	
☒	☐	ChIP-seq
☒	☐	Flow cytometry
☒	☐	MRI-based neuroimaging

Antibodies

Antibodies used	Anti-Glutathione Peroxidase 4 antibody, Rabbit monoclonal, Abcam catalog No. ab125066, 1: 200 diluted; Anti-4 hydroxynonenal antibody, mouse monoclonal, Abcam catalog No. ab48506, 1:50 diluted; Goat Anti-Rabbit IgG H&L (HRP), Abcam catalog No. ab205718, 1:1000 diluted; Rabbit Anti-Mouse IgG H&L (HRP), Abcam catalog No. ab97046, 1: 10000 diluted; HRP Anti-6X His tag antibody [GT359], Abcam catalog No. ab184607, 1:10000 diluted.
Validation	Antibody was validated by manufacturers (https://www.abcam.cn/products/primary-antibodies/glutathione-peroxidase-4-antibody-epncir144-ab125066.html; https://www.abcam.cn/products/primary-antibodies/4-hydroxynonenal-antibody-hnej-2-ab48506.html; https://www.abcam.cn/products/secondary-antibodies/goat-rabbit-igg-hl-hrp-ab205718.html; https://www.abcam.cn/products/secondary-antibodies/rabbit-mouse-igg-hl-hrp-ab97046.html) and titrated in our lab prior to use.

Eukaryotic cell lines

Policy information about cell lines and Sex and Gender in Research

Cell line source(s)	The HeLa cell line and Human lung cancer H1299 cell line were used.
Authentication	Human lung cancer H1299 cell line was obtained from National Collection of Authenticated Cell Cultures, Shanghai, China. The HeLa cell line (ATCC CCL-2) was obtain from ATCC.
Mycoplasma contamination	All cell lines were tested negative for mycoplasma contamination.
Commonly misidentified lines (See ICLAC register)	N/A

Animals and other research organisms

Policy information about studies involving animals; ARRIVE guidelines recommended for reporting animal research, and Sex and Gender in Research

Laboratory animals	Male BALB/C nude mice (5 weeks) were used in this study.
Wild animals	N/A
Reporting on sex	only male mice were used.
Field-collected samples	N/A
Ethics oversight	The animal experiments were performed according to the standards set forth in the Guide for the Care and Use of Laboratory Animals (National Institutes of Health, 1985). Experimental protocols were approved by the Animal Care Committee at College of Life Sciences of Shandong University (SYDWLL-2023-014).

Note that full information on the approval of the study protocol must also be provided in the manuscript.

Plants

Seed stocks	N/A
Novel plant genotypes	N/A
Authentication	N/A
